# Supporting Double Duty Caregiving and Good Employment Practices in Health Care Within an Aging Society

**DOI:** 10.3389/fpsyg.2020.535353

**Published:** 2020-11-11

**Authors:** Sarah I. Detaille, Annet de Lange, Josephine Engels, Mirthe Pijnappels, Nathan Hutting, Eghe Osagie, Adela Reig-Botella

**Affiliations:** ^1^Department of Human Resource Management, HAN University of Applied Sciences, Nijmegen, Netherlands; ^2^Department of Psychology, Universidade da Coruna, A Coruña, Spain; ^3^Faculty of Psychology, Open University Heerlen, Heerlen, Netherlands; ^4^Norwegian School of Hotel Management, University of Stavanger, Stavanger, Norway; ^5^Faculty of Psychology, Norwegian University of Science and Technology, Trondheim, Norway

**Keywords:** sustainable employability, double duty caregiving, self-management, HRM, scoping review, qualitative research

## Abstract

**Background:** Due to the aging society the number of informal caregivers is growing. Most informal caregivers are women working as nurses within a health organization (also labeled as double-duty caregiver) and they have a high risk of developing mental and physical exhaustion. Until now little research attention has been paid to the expectations and needs of double duty caregivers and the role of self-management in managing private-work balance.

**Objective:** The overall aim of this study was to investigate the expectations and needs of double duty caregivers in Netherlands, and to examine the meaning of self-management in managing work-life balance.

**Method:** Different research methods have been applied in this exploratory study. Firstly, a scoping review has been conducted on the topics self-management and sustainable employability of double-duty caregivers using the search engines: CINAHL, MEDLINE, PubMed, and Google Scholar. Furthermore, a qualitative study has been conducted through focus groups with double duty caregivers.

**Results:** Twenty studies that met the inclusion criteria (i.e., nurses with double duty caregiving tasks) could be identified. We found that double duty caregivers have different motivations for being a double duty caregiver based on internal and external expectations. Double duty caregiving causes a lot of mental and physical pressure for the caregiver. To be able to combine both duty’s, double duty caregivers need flexibility and understanding from the workplace. Through two focus groups (*N* = 17) we found that social support from the workplace is not enough to be able to manage the situation. Self-management skills are important to be able to communicate effectively with the workplace and community care organizations about the kind of support needed. Also, health care organizations should offer the same support to double duty caregivers as any other informal caregiver.

**Discussion:** Double-duty caregivers are at high risk of developing symptoms of overload and risk of reduced self-management quality and employability levels across time. Health care organizations and the double duty caregiver often wait too long to act instead of taking more preventive measures. Furthermore, community care organizations should dialog with double duty caregivers about their wishes concerning the division of caring tasks. This finding calls for special attention, with long-term solutions at both macro (health-care level), organizational (meso-level), and employee level (micro level).

## Introduction

According to data from World Population Prospects: the 2019 Revision, by 2050, one in six people in the world will be over the age of 65 (16%), up from one in 11 in 2019 (9%). By 2050, one in four persons living in Europe and Northern America could be aged 65 or over. In 2018, for the first time in history, persons aged 65 or above outnumbered children under 5 years of age globally (United Nations, 2019). The number of persons aged 80 years or over is projected to triple, from 143 million in 2019 to 426 million in 2050. Population aging is to become one of the most significant social transformations of the twenty-first century, with implications for nearly all sectors of society, including labor and financial markets, the demand for goods and services, such as housing, transportation and social protection, as well as family structures and intergenerational ties (United Nations, 2019).

Due to the aging population and decentralization of health care more people are expected to live at home as long as possible. Family members are taking on more frequent and complex are giving responsibilities than in the past. Many health care providers in western society are trying to balance family care with professional care responsibilities (Ontario Ministry of Health and Long-Term Care, 2014a; Ontario Ministry of Health, and Long-Term Care, 2014b).

In 2014 there were four million adults in Netherlands with informal caregiving tasks that amounted to more than 8 h a week (National Government, 2019). Informal care tasks include the help that people offer to relatives (e.g., family, friends) with health problems (De Boer et al., 2019). Informal care represents the most performed kind of long-term care throughout the world (Triantafillou et al., 2010) who conducted a European report on informal care, define informal caregiving as “care mainly provided by family, close relatives, friends, or neighbors” who are rather non-professional, do not have contracts concerning caring responsibilities, nor receive a salary for the care they provided. In the literature different reasons have been found for adopting informal caregiving roles. The most frequent reasons found in the literature are a sense of family obligations, driven by tradition and culture, moral values, and in many cases the issue of personal consideration, with focus on feelings of meaning, purpose and reciprocity was mentioned as reason (Chiao et al., 2015; van Groenou and De Boer, 2016). Tradition and cultural norms can motivate individuals to take on caregiving tasks even though they experience this as a burden (Sutter et al., 2016). Furthermore, as reported by Huber and Kuehlmeyer (2012), the decision to stay with and care for patients is not a voluntary one but depends on the type of responsibility in the relationship: asymmetric responsibility between a mother and a son and reciprocal responsibility between partners or friends. This means that a person becomes a caregiver unintentionally or without consciously choosing to be one. Therefore, the caregivers do not have the chance to negotiate about the conditions needed to become a caregiver and find it difficult to set boundaries afterward.

Across the literature, it is widely acknowledged that informal caregivers have a high risk of developing burnout and suffering from exhaustion as well as developing chronic physical complaints as caregiving tasks are stressful in combination with other tasks (Revenson et al., 2016; Gérain and Zech, 2020). Informal caregivers have been described as experiencing psychological, social, relational, emotional, and financial burden (Halpern et al., 2017; Wang et al., 2018). Increased levels of distress and anxiety are reported by half of the informal caregivers of patients with advanced-stage cancer (Goren et al., 2014; Moretta et al., 2014; Reblin et al., 2016; Rumpold et al., 2016; Gérain and Zech, 2019). The tasks of informal caregivers can be divided in three different groups: (1) Personal care or routine day living activities (e.g., bathing, toileting, eating, and dressing), (2) Household work (cooking, cleaning, and gardening), (3) Companionship, emotional support, and (4) economic support (Triantafillou et al., 2010; Heitink et al., 2017; Klages et al., 2020).

Within the healthcare and welfare sector, the ratio of informal caregivers is on average higher than in other sectors, namely one in four (Sociaal Cultureel Planbureau, 2019) and in recent research among nurses, 31% reported informal care tasks in addition to care tasks at work (De Lange et al., 2020). Different studies have found that women working in health care are more likely to be primary caregivers providing more direct care relating to ADL (Activity of daily living) of elderly care recipients than men (Navaie-Waliser et al., 2002; Killien, 2004). Often, nurses with informal care duties are exhausted at the end of the day and report a poorer self-rated health (DePasquale et al., 2016). Various studies also show that employees with informal care tasks have a lower work capacity than employees without these tasks (Mazanec et al., 2011; Heger, 2014). Providing informal personal care is significantly associated with poor mental health (OR = 1.23, 95% CI = 1.04–1.47) and poor physical health (OR = 1.18, 95% CI = 1.01–1.38). These factors can lead to prolonged sickness absence and turn-over in the health sector (Hiel et al., 2015; De Lange and Van der Heijden, 2016; Holland et al., 2019). Within the healthcare and welfare sector, various labor market trends and developments are on the way that have an impact on the labor force and quality of health care. Firstly, the sector is struggling with an aging workforce and hazing (too little inflow from younger employees); secondly, in 2018, 55% of employers in the care and welfare sector indicated that they had a shortage of staff in certain positions [an increase compared to 2016 (33%) and 2017 (45%)]; The demand for employees with competences for more complex care issues is increasing, the demand for low-skilled employees is falling; task shifts take place between the care professional and the caregiver; more than half (57%) of the care institutions use volunteers and informal careers for tasks that until recently were carried out by care professionals. As a result, healthcare and welfare institutions in Netherlands have to pay more attention to the quality of health care they can deliver and as well to the sustainable employability of their staff, in particular to their double duty caregivers (Josten and De Boer, 2015; Detaille et al., 2019).

In the context of double duty caregiving, sustainable employability means that nurses continuously have realizable possibilities in their working life as well as the conditions for being able to (continue to) function in current and future work while maintaining health and well-being (Van der Klink et al., 2010). This definition implies that nurses must be able to give direction to their sustainable employability potential (De Lange and Van der Heijden, 2016), but that is not easy when being a double duty caregiver as people with informal caregiving tasks in combination with a regular job have a higher risk of having stress, absenteeism and burnout (Plaisier et al., 2015; De Boer et al., 2019).

## Theoretical Framework

Different background theories have been used in this study as background for the scoping review and qualitative research. To begin with the Job Demands–Resources Model (JD-R; Schaufeli and Bakker, 2004; Bakker et al., 2005) whereby factors of job demands (perceived workload) and the ability to manage these (resources) analyzed. The theory states that job demands may become damaging when the demands and stressors associated with the job (perceived workload) are not in balance with the opportunity and ability to make use of resources. The support of the organization is crucial in supporting the workforce in achieving work- private life balance.

Another theory which has frequently been used in double duty caregiving literature is the impact of specialized knowledge on nurse family members. A study by Giles and Williamson (2015) has shown that many nurse family members feel a conflicting desire for healthcare personnel to know they are a nurse so that they will receive technical communication (Giles and Williamson, 2015). Some nurses want to be acknowledged and even respected in their role as professional caregiver (Giles and Williamson, 2015). This conflict of roles can reinforce the role of being a double duty caregiver in nurses.

Another theory to reinforce being a double duty caregiver is the job-control theory and the theory of conservation of resources theory (Hobfoll, 1989; Park et al., 2014). This hypothesis assumes people possess finite personal resources to distribute among their social roles, As people help others and manage these demands, they overload by placing themselves in high-demand why receiving, low-resource situations specially when caring for (sick) children or relatives.

Although informal caregiving is gaining importance within Healthcare specially in western countries, it is not clear which personal and external factors influence the motivation of becoming a double duty caregiver and influence the self-management of double duty caregivers in Netherlands. In this exploratory study we are firstly conducting a scoping review with the aim of describing the findings and range of research concerning the impact of double duty caregiving on the quality of life of the caregiver from the perspective of a nurse with secondary vocational education working at a middle or small hospital or daycare organization who combines work as a nurse and caregiving duty’s. Furthermore, we collect information from the literature about which personal and external factors influence the motivation and self-management of double duty caregivers.

Secondly, a qualitative study has been conducted aimed to answer the following research questions:

1. Which impact does double duty caregiving have on the quality of life of the caregiver (nurses working at two elderly health care organization in Netherlands)?
2. Which personal and external factors influence the motivation and self-management of double duty caregivers (nurses working at two elderly health care organization)?

To our knowledge, not many studies have combined both personal and external factors as well as how both factors interrelate with each other. This study is therefore contributing to create an awareness and possible solutions to this growing social issue.

## Materials and Methods

### Methodological Theoretical Framework

This research article consists of two exploratory studies which were conducted between January 2019 and November 2019. Firstly, a scoping review has been conducted to distillate the most important issues that concern double duty caregiving (nurses). The aim of the scoping review is to make a synthesis of the evidence of available information on this topic and to provide a broad overview of the available research evidence in order to explore a relatively new topic (Arksey and O’Malley, 2005). For the scoping review we have used a systematic review approach earlier described by Munn et al. (2018). The research strategy has been developed together with all the co-authors in collaboration with the librarian of the study center of the university. Different calibration sessions were conducted to define the search strategy, ensure consistency in the selection of articles and to improve inter-rater agreement. Furthermore, the different articles have been reviewed in two selection rounds by at least two authors and the themes have been analyzed by all the authors.

Secondly, we conducted a thematic analysis of qualitative data based on focus groups with nurses from two elderly health care organizations in the Netherlands to explore the different roles double duty care givers describe. Earlier research has shown that different caring roles can be found (Ward-Griffin et al., 2015; Klages et al., 2020). Previous research by Klages et al. (2020) has explored the blending of mothering and caregiving roles. In this current research we have explored different roles in double duty caregivers. The results of the scoping review have been used to create a sense of urgency form the perspective of the HR department and management from both elderly home care organizations to be able to conduct further research in the form of qualitative narrative research (focus groups). Furthermore, the results of the scoping review have been used to the construct the topic list for the focus groups as well as for the final synthesis of the results.

### Study 1: Scoping Review

A scoping review has been conducted (by JE, AD, EO, SD, and AR-B) to examine which personal (self-management) and work-related factors influence the sustainable employability (i.e., work ability, need for recovery, vitality, sickness absence, presenteeism, and work capacity) of nurses with double duty care tasks and which self-management strategies and HR instruments are used by nurses and organizations on improving sustainable work functioning. A scoping review is used when: (a) a narrow review question cannot be defined; (b) studies have employed a range of data collection and analysis techniques; (c) no prior synthesis has been undertaken on the topic; and d) the reviewers are not going to assess the quality of the studies reviewed (Crooks et al., 2010), The reason for choosing a scoping review is that until now no longitudinal cohort studies or effect studies have been carried on the sustainable employability of nurses with double duty caregiving tasks. The scoping review has been conducted by using the following search engines: MEDLINE, Pubmed, PsycInfo, Web of Science and BSC. The search strategy has been developed by all the authors in collaboration with the Study Center of the HAN University of Applied Sciences. The following keywords were used in this search: #1TS = [“nurse” or “nurses” or (“nursing” NEAR/1 (staff^∗^ OR professional^∗^ OR Assistant^∗^ OR aide^∗^ OR Auxiliar^∗^)) OR (“Home” NEAR/1 (Health^∗^ OR “care”) NEAR/1 Aide^∗^) OR (Physician^∗^ NEAR/1 (Assistant^∗^ OR extender^∗^)) OR Feldsher^∗^ OR (Auxiliary N1 Health N1 Worker^∗^)] #2TS = [(“work” NEAR/1 (abilit^∗^ OR capab^∗^ OR vitalit^∗^ OR capacit^∗^)) OR (“need” NEAR/1 “for” NEAR/1 “recovery”) OR ((sick^∗^ OR “illness” OR “disability”) NEAR/1 (absen^∗^ OR “leave” OR day^∗^)) OR presenteeism^∗^ OR absenteeism^∗^] #3TS = [carer^∗^ or caregiv^∗^ OR caretaker^∗^ OR custodian^∗^ OR guardian^∗^ OR (“care” NEAR/1 (giv^∗^ OR partner^∗^ OR taker^∗^)) OR (“family” NEAR/1 (obligation^∗^ OR “care”)) OR (“family” NEAR/1 burden^∗^) OR (“voluntary” NEAR/1 aid^∗^)] dual role/burden, dual caregiving roles, nursing/caregiving, nurse, nurse practitioner, health (care) professional, registered nurse, occupational/professional care, familiar/informal care, informal caregiving/caregiver, family caregiver, double duty caregiving, and multiple caregiving.

**FIGURE 1:**
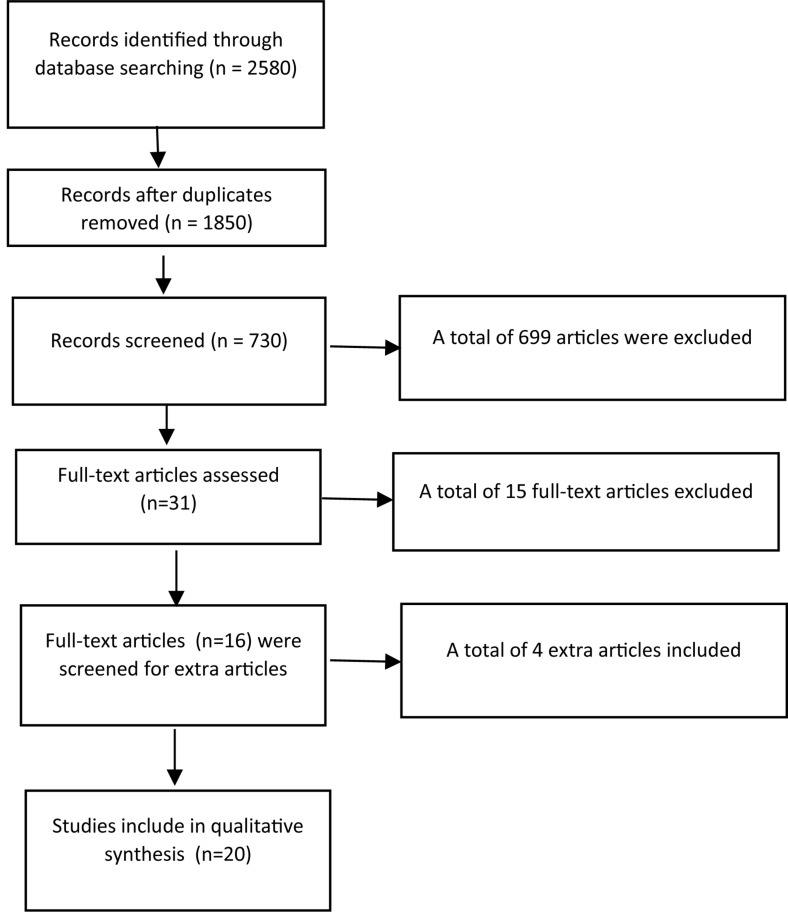
Prisma Chart of literature review.

Our scoping review focuses on double duty caregiving from the perspective of a nurse with secondary vocational education working at a middle or small hospital or daycare organization who combines work and caregiving duty’s. We have not included other highly educated health-occupations (like doctors or nurse-practitioners) and no work-family spill or effects of double duty caregiving and COVID-19 as subject. We have used the definition of caregiving as defined in the introduction of the article. Articles older than 1990 were not included in the review as well as articles not written in English or Dutch (Figure 1 and Table 1).

**TABLE 1:** Overview of included articles (*N* = 20).

Author	Year	Title	Method	Participants	Results/Themes
St-Amant	2014	Professionalizing familial care	Interviewed by telephone at multiple time points over a 6-to 12-month period.	*n* = 32 registered nurses	Quality of life is associated with Work-Family Balance (both Work-Family Conflict as Work-Family Enrichment).
Son	2007	The caregiver stress process and health outcomes	Qualitative (in-depth interviews + focus groups)	*n* = 234 primary caregivers of elderly relatives with dementia	Higher levels of both objective and subjective stressors were associated with all three dimensions of caregiver health: poorer self-reported health, more negative health behaviors, and greater use of health care services. The association between objective stressors and health was mediated by caregivers’ feelings of overload.
Ward-Griffin	2004	Nurses as caregivers of elderly relatives: negotiating personal and professional boundaries	Qualitative, descriptive, feminist narrative In depth-interviews.	*n* = 15 registered nurses of two community health-care agencies in southwestern Ontario	Findings revealed that double-duty caregivers are located at the juncture of public and private domains of caregiving, where they must constantly negotiate the boundaries between their professional and personal caregiving roles. The findings highlight the need to explore the interface between women’s family and work lives and the need for policies that promote the health of double-duty caregivers.
Ward-Griffin	2011	Compassion fatigue within double duty caregiving: nurse-daughters caring for elderly parents	Qualitative, secondary analysis Transcripts of in-depth interviews.	*n* = 20 female registered nurses, identified as “living on the edge”	Outcome is compassion fatigue; being both a nurse and an informal caregiver leads to the blurring of the boundaries between professional and personal care and ultimately to compassion fatigue (not the same as burnout).
Scott	2006	The impact of multiple care giving roles on fatigue, stress, and work performance among hospital staff nurses	Cross-sectioneel onderzoek: logboekjes per dag + VAS-schalen	(*n* = 393) hospital staff nurses	Fatigue and stress are significantly higher among nurses who care for both children and parents. Nurses who care for parents are more tired, have a greater sleep deprivation, and have a greater chance of making mistakes at work.
Gottlieb	1996	Predictor of work-family conflict, stress, and job satisfaction among nurses	Questionnaire and interviews	(*n* = 101) hospital-based nurse	Research into the extent to which work-family conflict (4 types) is predicted by insufficient support at home and at work, long hours, frequent changes in the schedule and a workload experienced as high (separately and in combination). And the effect of this on experienced stress and job satisfaction. Many links were found, but not those linked to informal care. Care for children does have a major influence. That is in line with the fact that they spend many more hours on children than on informal care.
Ruthman	1996	Caregiving as women’s work: women’s experiences of powerfulness and powerlessness as caregivers	Qualitative research	*n* = 5 nurses	In this study, two groups of women caregivers, older family caregivers, and women who do ”double duty” caregiving (paid health care professionals who simultaneously are unpaid elder caregivers in their “off” time), participated in daylong research workshops in which they first identified dimensions of an “ideal” caregiving situation and then, using a critical incident technique, explored the meaning of “power”-feeling powerful and powerless-for them as caregivers
Ward-Griffin, et al.	2005	Double-Duty caregiving: Women in the health professions	Narrative study	*n* = 37 (15 nurses, 6 physicians, 7 physiotherapists, 9 social workers)	Study findings suggest that female health professionals who assume familial responsibilities continually negotiate the boundaries between their professional and personal caring work. Despite the use of a variety of strategies for managing their double-duty caregiving demands, many women experienced a dramatic blurring or erosion of these boundaries, resulting in feelings of isolation, tension, and extreme physical and mental exhaustion.
Bouman and Dorant	2013	Double-duty caregivers: healthcare professionals juggling employment and informal caregiving. A survey on personal health and work experiences	Questionnaire	*n* = 45 low intensity informal caregiving *n* = 48 high intensity informal caregiving	Low-intensity group caregivers, finds it significantly more difficult to combine work and informal care, and experiences significantly more problems than High intensity caregivers due to sudden interruptions at work, although, in both groups, the percentage of caregivers reporting interruptions is very low (22% of the low- and 18,8% of the high-intensity caregiver group report being interrupted “monthly”).
Cortese, Colombo, and Ghislieri	2010	Determinants of nurses job satisfaction: the role of work–family conflict, job demand, emotional charge and social support	Questionnaire	*n* = 351 professional nurses	The findings suggest that there is a relationship between work-home interference and work-satisfaction.
DePasquale, Davis, Zarit, Moen, Hammer, and Almeida	2016	Combining formal and informal caregiving roles: The psychosocial implications of double- and triple-duty care.	Questionnaire	*n* = 1399 women working in nursing homes in the United States	Compared with nonfamily caregivers, double-duty child caregivers indicated greater family-to-work conflict and poorer partner relationship quality. Double-duty elder caregivers reported more family-to-work conflict, perceived stress, and psychological distress, whereas triple-duty caregivers indicated poorer psychosocial functioning overall. Discussion: Relative to their counterparts without family caregiving roles, women with combined caregiving roles reported poorer psychosocial well-being. Additional research on women with combined caregiving roles, especially triple-duty caregivers, should be a priority amidst an aging population, older workforce, and growing number of working caregivers.
Ward- Griffin, Brown, St-Amant, Sutherland, Martin-Matthews, Keefe, and Kerr	2015	Nurses Negotiating professional–Familial care boundaries: Striving for balance within double duty caregiving.	Two-phase mixed-methods study	*n* = 32 double duty caregivers	Participants in this study experienced conflict from being “torn between two worlds” as they negotiated their dual roles of professional nurse and family caregiver. The findings also suggest that lack of resources and support from the formal health care system contributes to the stress of caregiving, and over time, the health of double duty caregivers. Three types of specific double duty caregiving (DDC) prototypes have been distinguished; making it work, working to manage, living on the edge.
Heitink, Heerkens, and Engels	2017	Informal care, employment and quality of life: Barriers and facilitators to combining informal care and work participation for healthcare professionals.	Semi-structured interviews	*n* = 16 healthcare employees who combine work and informal care.	Respondents combine work and informal care because they have no other solution. The top three reasons for working are: income, escape from home and satisfaction. The biggest problems informal carers experience are a lack of time and energy. They are all tired and are often or always exhausted at the end of the day. T
Wohlgemuth	2013	Advantages and Challenges: The Experience of Geriatrics Health Care Providers as Family Caregivers	Semi-structured interviews	*n* = 16 (12 nurses, 3 physicians, 1 social worker)	The authors found 3 major themes: (a) dual role advantages and disadvantages, (b) emotional impact of dual roles, and (c) professional impact of family caregiving. Participants reported their own geriatrics expertise provided both advantages and disadvantages in caring for their older family members.
Denton	2002	Job stress and job dissatisfaction of home care workers in the context of health care restructuring	Multi-method study (survey + focus groups)	*n* = 892 employees, *n* = 99 (focus groups)	Focus group participants indicated that health care restructuring has resulted in organizational change, budget cuts, heavier workloads, job insecurity, loss of organizational support, loss of peer support, and loss of time to provide emotional laboring, or the “caring” aspects of home care work. Analyses of survey data show that organizational change, fear of job loss, heavy workloads, and lack of organizational and peer support lead to increased job stress and decreased levels of job satisfaction.
Ross, Rideout, and Carson	1996	Nurses’ work: Balancing personal and professional caregiving careers. Canadian	Interviews + diary	*n* = 40 full-time nurses	Most nurses experienced high levels of stress associated with caregiving in both their professional and private lives. The following themes emerged: an ethic of high expectation, feeling torn between two worlds, a sense of working in isolation, and working in overdrive. The rewards/benefits included remuneration, recognition and self -esteem, opportunities for personal growth, and opportunities for family growth.
Klages, East, Usher, and Jackson	2020	Modes of Informed Caring: Perspectives of Health Professionals Who Are Mothers of Adult Children with Schizophrenia	Interviews	Unknown	Different types of caring roles have been distinguished in this study; Monitoring of care, communication about care, physical aid of financial aid, professional knowledge and mothering.
DePasquale	2020	Family−supportive supervisor behavior positively affects work behaviour and non-work well−being among men in long−term care	Survey	*n* = 122 men working in U.S.-based nursing homes	Results from this study suggest that family-supportive supervisor behavior is an important workplace resource for the work behavior and family time adequacy of men in the long-term care workforce with additional non-work benefits for double duty caregiving men. Family-supportive supervisor behavior is a low-cost, modifiable workplace strategy that warrants consideration in efforts to attract and retain men in the nursing profession amid their increasing involvement in non-work care roles.
Gérain and Zech	2020	Do informal caregivers experience more burnout? A meta analytic study	Systematic review and meta-analysis	*n* = 17 studies	The present results support the increase of strain experienced when someone informally provides care to a relative. This was clearly shown for parents and formal caregivers, and to a lesser extent for spouses and white-collar workers
Jones	2020	A different point of view: The lived experiences of registered nurses as family caregivers	Dissertation	Mixed study method Literature review (*N* = 7), qualitative study (*N* = 25) and survey	Registered nurse family caregivers have unique characteristics, needs, and challenges that set them apart from layperson caregivers, but they can also provide a unique insight into patient safety that could lead the way to practical patient safety improvement initiatives.

### Data Extraction

A spreadsheet was created to chart the information that contributed to answering the research questions. Details of publication information, the aim and study sample were recorded. This process was carried out by SD, MP, NH, and AR-B. The information extracted that helped answer the research questions was discussed with the other authors during team meetings in order to work toward an overall perspective on the factors emerging from the literature. Disagreements were discussed until a consensus was reached. To chart the informational points extracted from the sources, a spreadsheet was created and securely hosted online that was used by all team members. Details regarding publication information, study design (if relevant), and the sample (if relevant) were recorded, along with any informational points pertinent to the overall scoping question. As discussed in the results section, four such themes were identified. Finally, the team then worked together to identify important avenues for future research by identifying knowledge gaps.

### Study 2: Qualitative Research

#### Data-Collection and Target Group

A qualitative study has been conducted on the basis of two focus groups (*N* = 17) with double duty care nurses. The participants were recruited from two elderly health care organization in the Province of Gelderland, Netherlands. The target population consisted of the recruitment process was organized through the department of HRM. The HRM department distributed the study information and recruited potential double-duty caregivers personally or through the newsletter of the health care organization. Potential participants responded to them. The HRM department was also responsible for organizing the focus groups and a lunch for the participants at the healthcare organization. At the end of the focus groups all participants received a small present to thank them for their contribution. The focus groups were conducted in Dutch, audio recorded and facilitated by the research team (NH, SD, and MP) by using a topic guide (Appendix 1). This guide was developed to improve understanding of double duty caregivers’ experiences which personal and external factors the need to be able to cope with the situation. Oral and written informed consent was obtained from all participants. Audio-recordings were transcribed verbatim by a transcription agency. The transcribed verbatim was sent to the participants for member check before the analysis.

### Data Analysis Method

The transcribed verbatim were reviewed in comparison to field notes obtained from the researchers (HN, SD, and MP). Thematic analysis was used for qualitative data analysis as it is a useful method to gain insight in the experiences of the participants (Clarke and Braun, 2018). A code list was generated based on the topic list of the focus group. Furthermore, open coding was used to code fragments which could not be framed by the code list generated from the topic list. Open codes were then discussed by the research team and new codes were added to the coding framework. Two members of the research team (SD and AR-B) independently coded the transcripts, reading and re-reading them to ensure familiarity with the data. Coding and analysis were done using ATLAS.ti software version 8, where segments from the data were copied and assigned to the generated codes. The qualitative data were presented in narrative form.

## Results

### Study 1: Scoping Review

Through the search strategy a total of 2580 were identified. BSC: 18 studies, Pubmed/Medline: 1244 studies, PsycInfo: 880 studies (50% overlap with Pubmed/Medline), Web of Science: 135, and google scholar 321 studies. After duplicate were removed a total of 1850 articles (abstracts) and 730 articles were screened in the first selection round on the following criteria: published after 1990, language English of Dutch, nurses caring for adults (elderlies), or/and relatives/friends had to be part of the sample, therefore, articles that did not have nurses as participants at all were excluded as well as work-family balance because of only childcare. The first and sixth author independently reviewed the abstracts, titles, and key words applying the above-mentioned inclusion and exclusion criteria. A total of 31 articles were selected for the second round. The first, second and third author independently reviewed the full-text articles applying the above-mentioned inclusion and exclusion criteria. After the second selection, a total of sixteen studies that met the inclusion criteria could be identified. Furthermore, we screened the literature list of the articles and obtained four extra articles for the review (see Table 1).

In the scoping review, 20 relevant articles were found focusing on the theme sustainable employability of double duty caregivers, being nurses. On the basis of the 20 relevant articles we have conducted a best synthesis of the literature on the sustainable employability of double duty caregivers. Different themes emerged from the literature review.

### Impact of Double Duty Caregiving

#### Personal Life and General Health

The literature study shows that nurses with double duty care tasks experience different challenges. Often, nurses with informal care duties are exhausted at the end of the day and experience a lack of time. Employees with informal care tasks also appear to have a lower work capacity than employees without these informal care tasks (Scott et al., 2006). Fatigue and stress are frequently reported in the literature. Some double duty caregivers may even be called triple duty caregivers, since there is also regular care needed for children or partners (Gottlieb et al., 1996; Ross et al., 1996; DePasquale et al., 2016). The consequences of double duty caregiving are for example, hobbies being scrapped, and social contacts are avoided, which means making the social network smaller and smaller. Double duty caregivers are often exhausted at the end of the day and experience a lack of time. This means that, for example, hobbies that take place in the evening are canceled and that social contacts are avoided, so that the social network becomes smaller and smaller. This leads to a poorer quality of life, stress, absenteeism and burnout (Heitink et al., 2017). Previous research has shown that more intensive forms of double care can have negative consequences for the (mostly female) nurses involved (Ward-Griffin et al., 2010), such as reduced chances of promotion, an increased chance of overloading and a reduced work capacity over the time (Boumans and Dorant, 2014).

### Motivation to Become a Double Duty Caregiver

#### External Expectations and Social Pressure

Furthermore, the article by Ward-Griffin et al. (2015) shows that nurses with informal care tasks experience different forms of social pressure, both internal pressure (from themselves), and external pressure (from others) to offer unpaid professional care in their personal lives to their loved ones. Denton et al. (2002) also found in their research that work-related stress was felt most acutely among nurses with informal care duties in managerial positions. The impact of dual care on a nurse depends on the expectations that there is in the family that one cares for each other and is also influenced by the support that the caregiver can engage on and off the job to cope with the care tasks to go. More and more caregivers provide long-term and intensive care, which can lead to a heavy emotional burden in addition to work.

Ward-Griffin et al. (2011) describe three types of double duty caregivers according to the experienced burden; “Making It Work,” “Working to Manage,” and “Living on the Edge.” In the “Making It Work” condition, caregivers were able to manage the experienced pressure themselves and to ask others for help to perform the dual care tasks. As a result, nurses with informal care tasks experienced minimal border blurring in care tasks at work and in private. The expectations for those who work to manage, “Working to manage,” were more burdensome and increased over time and the work-life imbalance too, with the risk of more complaints and work problems. The most burdensome double care condition was the “living on the edge” category of nurses. These nurses indicated that double care was too burdensome (too many expectations in work and private life both internally and externally and little to no support) and that they experienced many problems in physical and/or mental well-being and therefore had a risk of failure. Caregivers experience a high home-work interference which can lead to conflicts at work or at home (Boumans and Dorant, 2014).

#### Blurred Boundaries

A study by St-Amant et al. (2014), shows that nurses with informal care tasks often have to negotiate about the time they do or do not use in home care versus care at work that there are fewer aids in providing informal care at home versus at work and that nurses do not have desirable informal care working conditions that they cannot discuss or adjust (for example, more risk of too much physical overload at home in lifting or offering help to family members). It also appears that attention must be paid to the nature and intensity of the informal care to be provided. For example, there may be physical versus more mental forms of informal care. Mental forms of informal care include, for example, more affective support for the person requiring care (Son et al., 2007). Both types of care can be found at work and in private informal care. As a result, the nurse experiences different support needs and has to learn to balance the care roles at work and in private in different ways (Ward-Griffin et al., 2010). Research by Ward-Griffin et al. (2010) shows that female nurses with dual care duties continuously negotiate the boundaries between their professional and personal care duties. Despite using a variety of strategies to manage their dual care, many women experience dramatic blurring or erosion of these boundaries, resulting in feelings of isolation, tension, and extreme physical and mental exhaustion.

### Self-Management Coping Strategies

#### Social Support at the Workplace

A number of resources are named in the literature to deal with the dual care tasks. Cortese et al. (2010) have investigated the extent to which work-family conflict is predicted among nurses with informal care duties due to the lack of support at home and at work. Long hours, frequent changes in the schedule and a workload experienced as high (separate and in combination with informal care tasks and care for the children) and the effect of these factors on the experienced stress and job satisfaction of nurses with informal care tasks. It is recommended to provide nurses with informal care tasks with social support at work, to participate in scheduling and to help them take self-management (setting boundaries and defining tasks). It is also recommended to support nurses with dual care tasks to take good care of themselves in terms of mental and physical health (taking adequate rest, getting help at work and paying attention to a healthy lifestyle) as well as encouraging double care tasks possibly separate or support with resources at work to keep them manageable (Rutman, 1996; Ward-Griffin, 2004). Double duty care workers tend to want to solve a lot themselves, precisely because they know how to perform care tasks, whether formal or informal. Earlier research indicated that nurses generally want to keep their own control over the balance in their lives, and that the work-life balance needs support (Heitink et al., 2017) when informal care tasks start to ask too much.

#### Making Use of Resources

According to Ward-Griffin et al. (2010, 2011), nurses with dual care duties must use negotiating strategies for this at work (for example, adjustments to rosters to be able to provide private care), but also have a conversation with the person asking for care at home (when work is not adapted) for example, to use more home care in private). These findings suggest that women with dual care duties, especially those with limited time, finances or other tangible support, may experience poor health over time. Ward-Griffin (2004) found that female community nurses caring for elderly family members used a variety of coping strategies, such as setting limits on the specific care they provided. Although women in the health professions are often viewed as needing to develop coping strategies to address the stress of “balancing” or “juggling” two or more roles, attempts at setting limits often prove unsuccessful in dealing with the demands and tensions of family caregiving (Ward-Griffin, 2004; Ward-Griffin et al., 2005). These strategies acknowledge and address the declines and losses which occur; examples include modifying behaviors, the use of external aids and activating unused resources (e.g., help from others).

Managing the challenges of Double Duty Caregiving is a demanding task that can be handled best with support from the environment. Research has shown that managers play an important role in supporting nurses with informal care tasks (Ward-Griffin, 2004). However, caregivers appear to find it difficult to start a conversation with their supervisor (Heitink et al., 2017). The nurses of the studies received support from different sources: from their families, especially spouses, siblings, or adult children (Ward-Griffin et al., 2005), and from their colleagues who covered shifts for them, changed their working schedules to help out or assisted them with their professional knowledge (Ward-Griffin et al., 2005; Wohlgemuth et al., 2013). Participants who had an understanding manager felt blessed Flexible working shifts, unpaid leave of absences and working part-time were supportive as well (Ward-Griffin, 2004).

In summary, the impact of caregiving on the sustainable employability of the double duty caregiver depends on:

a) The own expectations that the double-duty caregiver has with regard to their own role as caregiver;
b) The expectations that exist in the family toward the double duty caregiver;
c) The experienced support that the double duty caregiver has at work;
d) The self-management skills of the double duty caregiver to deal with the care tasks and organize support at home and at work.

### Qualitative Research Results

#### Exploring the Motivation of Double Duty Caregiving

Some double duty caregivers claim that their motivation to be a double duty caregiver is that they are “the only one in the family” who can take up this caring task as nobody else in the family can manage this due to their specific knowledge and skills in healthcare. This results in high expectations of self, as well as expectations from family and other health professionals, to provide “nursing” care.

*“I’m the only daughter. I do have four brothers, but preferably it is “our daughter”. So yes, that has been and is intense. Well, caregiving has grown a lot, because I do everything. I bring the laundry. I visit her every day. I do the shopping, the laundry, every day. If the alarm goes off, I will pick her up again. That’s how it turned out to be a lot” (nurse, age range 55–60 years)*

In addition to their own expectations of caregiving, participants also spoke of their moral duty to take caregiving tasks over as the workload of nurses in elderly care homes is high now and there are not enough nurses at the workplace to deal with all the caregiving tasks. They realize that caregivers are needed nowadays in order to manage taking good care of elderly people living at home. Double duty caregivers realize that double duty care giving is a specific 21th century problem as women now a day’s work and women mostly take up the role of caregiver within a family.

*“Well, do you know what the problem is? Nowadays. In the workplace we have less time for people, so the family has to help. Caregivers must come. But we are the people who work in the workplace and we are also the caregivers. Yes. That wasn’t before. In the old days, forty or 50 years ago, the women did not work, and they had time for those things. Yes, well, it is true”. (nurse, age range 45–55 years*)

External expectations often position participants in difficult situations, such as the one below in which the nurse worked as well as being caregiver in order to help out “colleagues in health care.” Nurses who take up double duty caregiving tasks realize that nurses nowadays have less time to complete their care tasks due to overload at work. Furthermore, nurses have a strong sense of responsibility to care for other people as they feel that nobody else will do it. This puts a high pressure on themselves to take on caregiving responsibilities than they can hardly manage.

*“Many services are being cut by the government nowadays like residual assistants, housekeeping, technical assistance. you can’t believe this! Healthcare is being cut down! This puts extra work and stress on family members like us, I think that if we as an informal caregiver were not there, a lot of people would end up very badly” (nurse, age range 50–55 years)*

*“We must both work and we must be caregivers and we must also be volunteers for anything and everything. You just have so many tasks. But yes, that is also the time of today. You are expected to take on these tasks. They find it very normal” (nurse, age range 60–65 years)*

*“Yes. I also find it quite intensive here. Almost nobody comes. Then I always think “I have to go and take a look again” (nurse, age range 40–45 years)*

An extra pressure to become double duty care worker is derived from the fact that not enough support is received from the *community care organization* day care organizations who usually provide this type of care for elderly people still living at home. Furthermore, day care organizations slightly decrease their control when they found out that the double duty care giver can take up the nursing tasks at home even if the double duty care givers asks for help and does not have all the materials at home to take up the caring tasks in an appropriate way.

*“Do you know what I really should like to change in health care? Well I didn’t always feel taken seriously by the community care organization. At some point they think that as a double duty caregiver you can do so much that you have to do it all by yourself for a while. I have had a situation; the skin from my mother was completely rolled up here. So, I said “madam has fallen out of bed and she has a wound so and so is the case, what should I do with it?” “Yes, put a gauze on it. They said. I said I can’t put a gauze on it, I won’t get it off the wound the next morning*…. *The community care organization said well, it doesn’t matter, you don’t have any other materials in the house, just do it”. So, I did and indeed, it has a hell in the morning taking the gauze off. I felt guilty about that as a caretaker at the time” (nurse, age range between 50–55 years)*

Double duty caregivers also encounter emotional problems if they can’t offer the type of caring aid needed. As they have more knowledge of health care than the average caregiver, they can’t conform themselves to non-professional caregiving. This can put an extra mental pressure on them and give double duty caregivers the feeling that they can’t deliver good quality care and that they are not taken seriously by the neighborhood caring organization.

*“There have been many situations at night that I was not taken seriously by the community care organization. That contributes to the fact that at a certain point you will say “I will not go that far anymore” (nurse, age range 55–60 years)*

The extra pressure that double duty caregivers experience, obliges them to set up boundaries and not go as far as they would like to go as they can’t deliver professional care in a home care environment.

#### External Expectations and Community Care Organization

Double duty care givers feel an external pressure from community care organizations to take more tasks up than they would like too. Especially when community care organizations find out that the care giver is a nurse then they give the double duty care giver more responsibility to take tasks over. Sometime double-duty caregivers are expected and allowed to take complex (clinical) tasks over even if they do not have the license to do this.

*“My mother also lived completely independently. She then received care from a community care organization. At some point, agreements were made about “they do this, and I do this”. The last 2 weeks I took care leave from my work to be able to take care of my mother. Then the daycare organization said “well, her daughter who is a nurse is here, so we don’t have to come.” (nurse, age range between 45–50 years)*

*“I had the same situation with my husband. He stayed at hospital for a year and when he came home, I just learned all the technical nursing procedures from at the hospital, because I was going to nurse and care for him at home. I took on complex nursing tasks that I don’t even have a license for as a nurse. I took Intensive Care tasks like placing a vacuum and I did everything. because you are a family member and a nurse, they let you do this” (nurse, age range 50–55 years)*

The communication and collaboration between community care organizations and the double duty caregiver does not always go well. Due to a lack of communication about mutual expectations, the double duty caregiver might feel a lack of support. On the other, double duty caregivers find it hard to set-up their boundaries and communicate their needs with the community care organization.

*“If they want good informal care, we must be taken seriously. And if an informal caregiver indicates “I can’t take it anymore”, they have to look for temporary solutions. I have not yet experienced that they looked for solutions. (nurse, age range 55–60 years)*

#### Self-Management of Double-Duty Caregiving

In order to manage additional caregiving demands, there should be a good relationship and open communication with the *community care organization* about mutual expectations concerning the caring tasks of family members. Because the high sense of responsibility is high double duty care givers might get carried away taking up tasks that are not the responsibility of a normal informal family caregiver. Also, double-duty caregivers struggle with defining their caregiving tasks. They feel highly responsible for the wellbeing of their family member and as they are a professional nurse, they struggle with which tasks a normal to take up and which tasks belong only to the community care organization. In this sense they would like to be informed better by the government or community care organization in the form of a manual or guide.

*“Well, I don’t know, but maybe it would be nice to have a manual on the extent to which family members help with feeding and everything, swallowing problems. May they be help everywhere or only with certain tasks added or will we not be left in a room with limited incentives? Don’t you think?” (nurse, age range 45–50 years)*

Others stand up for themselves and set up their own boundaries by not taking any care tasks over that they do not feel comfortable with. The consciously have to make a choice about which tasks they want and do not want to take and communicate this effectively to the community health organization.

*I have been my mother’s caregiver for 7 years. That was also quite intense. Daily. My mother lived in a sheltered home outside of the city. I cleaned there, did the laundry. No care duties. I consciously chose that. I said, ”I don’t want that, I don’t”. (nurse, age range 50–55 years)*

At the workplace double duty care givers do not feel that they receive enough support at work even though they acknowledge that colleagues and supervisor were identified as the primary source of support needed to be able to cope well. Nurses felt an extra pressure from the workplace to keep on going as the could not be replaced by another staff member. Due to the current shortage of health professionals’ supervisors and colleagues projected a moral pressure on double duty care givers to not take sick leave or care leave.

*“My son had surgery, so I was a caregiver. I had a lot of stress, but I wasn’t allowed to get sick. ”Oh, Francoise, please don’t get sick, don’t you get sick, right?” So, went on and on until half December I was so tired that I collapsed and went home crying! The next day my supervisor called me “Would you please, come and work if only for 2 h we need you.” (nurse, age range 40–45 years)*

“*Same here. I had conversations with my supervisor and day coach “Please don’t you get sick, you get 2 days off from us now and don’t get sick, because we can’t take it, because you can’t be replaced.” I said “well, let’s then take a look.” I mentioned it, right? I rang the bell myself. I haven’t heard from my supervisor anymore, until I thought “let’s just ring the bell, boys, what are we going to do with this?” This is not right!” (nurse, age range 45–50 years)*

Double duty care workers do not feel supported by their supervisors and health care organization to be able to combine informal care with work. The problem of double-duty caregivers is not affronted as a “work problem.” Conversations about how to prevent presenteeism and long-term sickness absence are not being held or not on a regular basis. As a result, the double duty care giver ends up exhausted or falls out which could be prevented.

*“In the meantime, I was working again two, 3 h a day, a few days later 15 h a week and now again 32 h a week*…*And at some point, I had something like “yes, but this is not going well at all”. My daughter came to live at home again, my grandmother became very ill. My grandmother died and I called in sick. I have had the greatest misery. They told me to come in the next day to talk and they told me that they would immediately place me in another department*…*.I was not amused!” (nurse, age range 55–60 years)*

The erosion of personal and professional caregiving boundaries usually occurred during a crisis or over time when the caregiving expectations and demands outweighed the available resources at the workplace. Combining both tasks feels as juggling.

*“How did I do that? How did I do it all? Combining work with private life? That’s just juggling, right? Just keep going on and on*…*.Yes, continue. That went very well. Until my grandmother died and then the bucket was full. Then they find it very strange at work that I need to take temazepam and oxazepam prescribed by the doctor. They find that very strange at work*…*” (nurse, age range 30–35 years)*

#### Knowledge and Skills to Cope at Work

Double duty care givers acknowledge that they do not always have the knowledge and skills to come up for themselves, set boundaries and negotiate with health care organizations about the distribution of caring tasks.

*“I think every double duty care giver needs to follow a self-efficacy training about how far I want to go. And also, how can I come up for myself, what are my rights at work and how can I communicate effectively and setup my boundaries at work” (nurse, age range 45–55 years).*

A common dilemma is how far do they can go and want to go. Double duty care givers do not always have the skills to communicate and negotiate effectively with their environment.

*“Yes, that is of course also different for everyone. How far do you go and how far do you not go? But if you say, “I don’t want to go further than this,” then they have to take that into account, “We won’t go further than that! This is important but they don’t do this, they don’t support us!” (nurse, age range 50–55 years)*

*“Well in my situation it was different. That was different for me, because I wanted to do that. Do you understand? Wanted to do that, right? Then it’s different. But yes, you must set up your boundaries and maintain that limit because otherwise you will also make mistakes*…*”(nurse, age range 55–60 years)*

#### Desired Support From the Health Care Organization

Double duty caregivers have different support needs in order to remain employable. Some double duty caregivers realize that the need support from the workplace to be able to cope with the situation. They also realize that they need to take up a healthy lifestyle with enough physical movement and relaxation to be able to stay vital at work. The following support wishes in relation to dual care emerged in the interviews:

*“I would like to receive support on how can I discuss my heavier informal care tasks with my supervisor and how can I be supported by my employer? I really do not know how ask for support and what the role of HR is in my organization. I have the feeling that they do not listen and care about us” (nurse, age range 50–55 years)*

*“On my days off I spend time with my mother. So how can I stay mentally and physically fit while having less and less time to relax or to participate in sports activities through my caregiving tasks? we just have to go on and on*…*I don’t see a solution” (nurse, age range 50–55 years)*

Nurses with informal care tasks tend to want to solve a lot themselves, precisely because they know how to perform care tasks, whether formal or informal. Earlier research indicated that nurses generally want to keep their own control over the balance in their lives and the work-life balance but do need support when the family caregiving tasks start asking too much.

## Summary and Discussion

The results of the scoping review and focus groups with double duty care nurses acknowledge that;

• Dual caregiving tasks are needed in order to keep the quality of health care for their family members acceptable. They feel obliged by their own expectations, the expectations of relatives and the expectations of the community care organizations to take on caregiving tasks.
• Experience difficulties in managing double duty caregiving tasks with private and work tasks.
• Experience distance from their health organization to combine double duty caregiving tasks.
• Find it difficult to cope with double duty caregiving due to fatigue and experience insufficient rest during the week to be able to recover.
• Some double duty caregivers take antidepressants to keep on going!
• Find it difficult to ask for social support and find solutions but acknowledge that self-management skills are important to cope with the situation.
• Experience insufficient attention to work-life balance at work.

The impact of caregiving tasks on the sustainable employability of nurses depends on their own expectations regarding their own role as caregivers; the expectations that exist in the family that one takes care of each other and the experienced support that the caregiver receives on and off work. The results of this study correspond with other studies related to informal caregivers in different domains. There are different motivations found in the literature and qualitative research for becoming an informal caregiver like a sense of family responsibility, tradition and cultural values and feelings of meaning, purpose and reciprocity. In the literature other similar reasons have been found. A study by Leonardi et al. (2017) found that caregivers take care of their relatives for different reasons. Among them, caregivers reported that they thought they could do it better than everybody else and, secondarily, because there were no other people that could do it or because others had no time available. In this study nearly all nurses reported being the only one in the family who could take this task on as they had the knowledge and skills to do so. The results of this study show the seriousness of the problem of dual function caregivers within health organizations. A study by Gérain and Zech (2019) provides strong indicators that informal caregiving has an impact on the development of burnout. It thus calls for a necessary consideration of informal caregiving burnout in itself (Gérain and Zech, 2019). The results allow the formulation of preventive measures in health organizations and improve the health of women caregivers. It is a pioneering study in Netherlands and will be complemented in the future with quantitative research and an intervention study. This study show that the problem of double duty care givers should be approached from a multi-level perspective (personal, business, health organizations, and health). The scoping review was carried out to explore a variety of themes concerning the impact of double duty caregiving on the quality of life of the caregiver as well as which personal and external factors influence the motivation and self-management of double duty caregivers.

**FIGURE 2:**
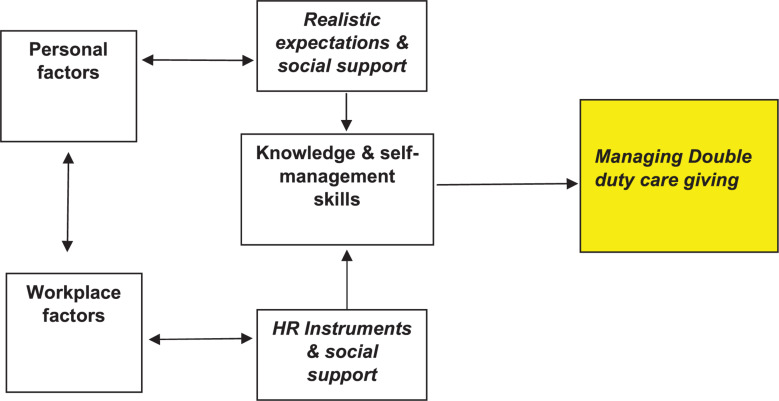
Conceptual model for future research on the sustainable employability of double duty caregivers.

In order to support the sustainable employment of double duty caregivers, it is recommended to use different HR instruments at work, but also to support nurses with dual care tasks in their own self-management (for example, when setting limits, and defining tasks). It is also recommended to encourage these nurses to take good care of themselves in terms of mental and physical health (taking adequate rest, seeking help at work and paying attention to a healthy lifestyle. The dialog with the supervisor appears to be of great importance and a possible key to identify (health) risks early and to be able to offer customized solutions. Possible strategies for reducing stress are, for example, strengthening one’s own management, increasing the leeway that work, the social environment and private life offer, and taking one’s own initiative to make informal care negotiable. Nurses can work independently on their self-management but can also be supported through Human Resource Management (HRM) with various types of support interventions. Self-management programs to stimulate the self-efficacy of the double duty caregiver could be offered at the workplace as well as a dialog tool that managers can use to assess the kind of support that double duty caregivers needed. Managers can also offer support by entering a discussion about which HRM interventions are on disposal or develop HRM interventions at the workplace to keep nurses permanently employable. On the basis of this study we have developed a theoretical model than can be used in future longitudinal research or effect evaluations of interventions (Figure 2 and Table 2).

**TABLE 2:** Adapted HRM bundles for workers with double duty care tasks.

Bundle HRM practice	Development	Maintenance	Utilization	Accommodative
Specific practice examples	Training Career development Promotion	Flexible working conditions like flexible time schedule. Exchange schedule with colleague Shorter workweek Ability to take days off when needed	Task enrichment Participation in decision making Horizontal job change Second career	Extra leave Sabbatical (Pre) pension Demotion Exemption from overtime Part-time retirement
Underlying purpose	Appreciation at work	Achieve a balance in managing double duty caregiving and preventing short-term sickness absence	Becoming more employable within and outside healthcare institutions	Recovering from overload double duty caregiving and preventing long-term sickness absence

## Limitations and Future Research

However, two main limitations exist. The first is that it was difficult to find through the search all the existing articles on the theme quality of life and sustainable employability of double duty caregiver nurses. We have specially focused on double-duty care nurses as we believe that the working conditions and social of nurses support at work is a significant problem to tackle within health organizations due to the labor market shortages. The combination of the search terms double duty care giving AND nurses also led to a huge amount of irrelevant articles about nurses delivering health care to patients and their relatives or health care programs for caregivers delivered by nurses. Furthermore, only English-language sources were retrieved and reviewed. No doubt there is literature in other languages which has not being considered in this review. The nurses concerned in this study (focus groups) represented a relatively small sample of 17 nurses working at two elderly care organizations in Netherlands from the same region in Netherlands. The number of cases could be increased and include also males instead of women. Even though it was already quite a problem to include 17 nurses due to the heavy workload and commitment of health-organizations. However, in the literature the number of recommended focus groups depends on the complexity of the study design and the target sample’s level of distinctiveness. A study by Cleary et al., 2014 has found that most studies include two focus groups. Studies with more than 3 focus groups are rarely conducted in the social sciences. In this study, recruitment proved to be difficult in practice due to working schedule times and informal care responsibilities. Furthermore, the recruitment process proved to be difficult due to work time schedules. Some double duty caregivers even managed to come to the focus groups on their day off. This might imply that a highly motivated group attended the focus groups as well as the group that have already find ways to cope with the situation. Some participants could only participate on their day off. The results of this might not be representative for all double duty caregivers in Netherlands like for example male double duty caregivers. A study by DePasquale (2020) suggests on the basis of other studies that male double duty caregivers risk less support at the workplace and more organizational penalties, face gender-related stigmatization and are perceived as less masculine by male and female peers (Ewald et al., 2020). In the future it would be interesting to conduct a more qualitative research among different types of double duty care givers but also a quantitative research like the study of Gérain and Zech (2020) which made use of a longitudinal cohort study to be able to validate our conceptual theoretical model. Furthermore, also a meta-analysis could be used in order to verify the results of our traditional review.

The scoping review approach has several limitations. Scoping reviews do not formally evaluate the quality of evidence and often gather information from a wide range of study designs and methods. By design, the number of studies included in the review process can be sizable. Thus, a large study team is typically needed to screen the large number of studies and other sources for potential inclusion in the scoping review. Because scoping reviews provide a descriptive account of available information, this often leads to broad, less defined searches that require multiple structured strategies focused on alternative sets of themes. Hand searching the literature is therefore necessary to ensure the validity of this process. Scoping reviews do not provide a synthesized result or answer to a specific question, but rather provide an overview of the available literature. Even though statements regarding the quality of evidence and formal synthesis are avoided, the scoping review approach is not necessarily easier or faster than the systematic review approach. Scoping reviews require a substantial amount of time to complete due to the wide coverage of the search implicit in the approach.

## Practical Implications

### Workplace and HRM Practice

The aforementioned results from the literature and qualitative research among double duty care givers showed that a diversity in type of support needs mentioned by caregivers in relation to self-management and sustainable employability at work and that customization is desirable, whereby not only curative but especially more preventive self-management based solutions are sought to help an overburdened double duty caregiver to remain employable by offering emotional and psychological support. Creating an understanding working environment, is important to enable double duty caregivers to remain employable. Employers and HR managers should converse with double duty caregivers in their workforce about familial care expectations, the resources they are using/needing, as well as how their caregiving role is impacting their labor force participation and health status. Double duty care givers can work independently on their self-management but can also be supported through Human Resource Management (Guest, 2011) with various types of support interventions. Previous research has shown that organizations can offer meaningful bundles of HRM interventions to keep nurses permanently employable (De Lange et al., 2015; Pak et al., 2019; see Table 2), namely:

1. Developmental HRM practices (or career policy), such as training and promotion, to help nurses with informal care tasks achieve higher levels of functioning and continue to develop;
2. HRM practices aimed at maintaining work capacity, such as job safety and flexible working hours (or working conditions policy), enabling workers to maintain their current level of functioning;
3. HRM practices aimed at protecting nurses through the protection or saving of nurses with informal care tasks (through demotion or task reduction). These HRM practices help overburdened nurses with informal care duties to function well at lower levels when retention or recovery is no longer possible;
4. HRM policy aimed at utilizing the existing experience, knowledge and skills of nurses, such as horizontal job changes or job enrichment, and using previously unused knowledge and skills of the nurse through starting a second career.

### Healthcare Providers

The findings of the literature and qualitative data have shown that double duty caregivers are confronted with different challenges compared to non-professional caregivers. This results in various implications for the health care providers who assist the relative of the double duty caregiver:

1) Healthcare providers should not assume that all nurse family members have the same expectations of the level of involvement in care and type of care they are willing to give. Mutual expectations should be communicated with each other at the start of the caring process.
2) The assessment of the desired level of involvement, skills and possibilities of the double duty caregiver is crucial to build a good relationship between the healthcare provider, the double duty caregiver and the patient.
3) Healthcare providers should acknowledge the double role of double duty caregivers and be aware of possible role conflicts.
4) Healthcare providers therefore can support them by offering consultations to identify possible conflicts and how to resolve them as well as empowering double duty caregivers.
5) Double duty caregivers must be continuously reminded by healthcare providers that they must take time for themselves and to not take more tasks up than needed.

Overall, it would be recommendable to health care organizations to assess how many double duty caregivers they have and to assess the work-ability and needs of these workers in order to detect possible conflicts in the work-life balance at an early stage. This will allow managers to take action on time aimed at preventing possible drop-outs and sickness absence as well as nurses leaving the workforce. Moreover, it appears that the complexity of double duty caregiving means that a “one size fits all” approach will not work to support nurses with informal care tasks.

## Data Availability Statement

The datasets generated for this study are available on request to the corresponding author.

## Ethics Statement

Ethical review and approval was not required for the study on human participants in accordance with the local legislation and institutional requirements. Oral informed consent to participate in this study and to report the findings in publications was provided by the participants’ (nurses) and the staff of the health care organization were the participants work before the start of the focus group. The oral informed consent has been recorded and transcribed.

## Author Contributions

SD, AL, JE, EO, NH, and AR-B: conceptualization. SD, AL, JE, EO, NH, and AR-B: methodology. SD, MP, and NH: data-collection. SD, MP, and NH: formal analysis. SD, AL, JE, EO, NH, MP, and AR-B: writing – original draft preparation. SD, AL, JE, EO, NH, MP, and AR-B: writing – review and editing. All authors have read and agreed to the published version of the manuscript.

## Conflict of Interest

The authors declare that the research was conducted in the absence of any commercial or financial relationships that could be construed as a potential conflict of interest.

## Appendix 1

### Topic List Focus Groups Double Duty Caregivers

#### Introduction

1. Brief introduction to research and focus group
2. Brief explanation of working method focus group (state of affairs, recording of conversation, confidentiality, etc.)

#### Opening Questions

1. Proposal round: name, profession/work, family composition/home situation, and possibly. Informal care tasks (including the type of informal care tasks and the number of hours per week).
2. What gives you the most satisfaction in your life (both work and private)?
3. What does a lot of energy currently cost you in your life? What is your current tax status?
4. Why did you decide to participate in this focus group? What do you expect from your participation?
5. To what extent does your private situation affect your work?
6. To what extent does your work affect your private life?
7. Did you tell at work that you are doing informal care tasks (or in the case of no informal care tasks: Is your work aware of the influence of your private situation on your work or the influence of your work on your private life?)

#### Experiences at Work

1. To what extent are you currently able to perform your work (activities) as you would like? How does the current situation relate to the past? (Eg when you were not yet an informal caregiver, when you were younger, when your private situation was different, etc.).
2. Which factors influence whether or not you can perform your work (activities) as desired?
3. Which problems at work do you encounter? Do you experience burdensome factors during work? (Consider time tax/problems, physical stress/problems, emotional stress/problems, social stress/problems, and mental stress/problems).
4. What consequences do these problems/stressful factors have for you (loss of work, not being able to finish work, physical or psychological problems, and leaving work to colleagues/asking for help from colleagues).
5. How do you deal with these problems and burdensome factors? (What has effect, what has no effect? Are you proud of certain things that you have addressed?).
6. What is the problem with performing informal care tasks and/or in your private situation, whether or not in relation to your work (activities) and the desired lifestyle and activities?
7. Do you experience sufficient support in the field of work, informal care tasks, and/or private matters? If so, from whom? So, no what consequences does that have for you?

#### Desired Situation

1. What would the desired situation with regard to your work (duties), informal care tasks, and further private life ideally look like?
2. What specific matters would you like to change with regard to your work (duties), informal care duties, and further private life?
3. What do you need to make these changes? Which support would be desirable in this regard (e.g., from the organization – HR, supervisor, and colleagues), so that you can better deal with your problems? What role do you play in this?

#### Preconditions

1. Would you like to learn how to better deal with your problems at work and at home?
2. If so, what would you like to learn/which topics are important?
3. How could a program support you in changing specific matters?
4. What form (online/offline/combination) do you think such a program should take?
5. What preconditions should a program meet before you participate in that program (size, duration, time, day, user-friendliness, interaction, tailoring, and support)?
