# Corrigendum: Supporting Double Duty Caregiving and Good Employment Practices in Health Care Within an Aging Society

**DOI:** 10.3389/fpsyg.2021.666611

**Published:** 2021-04-29

**Authors:** Sarah I. Detaille, Annet de Lange, Josephine Engels, Mirthe Pijnappels, Nathan Hutting, Eghe Osagie, Adela Reig-Botella

**Affiliations:** ^1^Department of Human Resource Management, HAN University of Applied Sciences, Nijmegen, Netherlands; ^2^Department of Psychology, Universidade da Coruna, A Coruña, Spain; ^3^Faculty of Psychology, Open University Heerlen, Heerlen, Netherlands; ^4^Norwegian School of Hotel Management, University of Stavanger, Stavanger, Norway; ^5^Faculty of Psychology, Norwegian University of Science and Technology, Trondheim, Norway

**Keywords:** sustainable employability, double duty caregiving, self-management, HRM, scoping review, qualitative research

In the original article, there was an error. A citation was not formulated in the correct way. A correction has been made to *Materials and Methods, Methodological Theoretical Framework, Paragraph 2*. The corrected paragraph is shown below.

Secondly, we conducted a thematic analysis of qualitative data based on focus groups with nurses from two elderly health care organizations in the Netherlands to explore the different roles double duty care givers describe. Earlier research has shown that different caring roles can be found (Ward-Griffin et al., 2015; Klages et al., 2020). Previous research by Klages et al. (2020) has explored the blending of mothering and caregiving roles. In this current research we have explored different roles in double duty caregivers. The results of the scoping review have been used to create a sense of urgency form the perspective of the HR department and management from both elderly home care organizations to be able to conduct further research in the form of qualitative narrative research (focus groups). Furthermore, the results of the scoping review have been used to the construct the topic list for the focus groups as well as for the final synthesis of the results.

The authors apologize for this error and state that this does not change the scientific conclusions of the article in any way. The original article has been updated.
